# Temporal Changes in Nitrogen Pollution in Northeastern Estonia

**DOI:** 10.1100/tsw.2001.304

**Published:** 2001-11-21

**Authors:** Valdo Liblik

**Affiliations:** Tallinn University of Educational Sciences, Institute of Ecology, North-East Estonian Department, Pargi 15, Jõhvi 41537, Estonia

**Keywords:** nitrogen oxides, sulphur dioxide, emission, air pollution, deposition load, critical levels, oil shale industry

## Abstract

During the last 5 decades the northeastern part of Estonia (the region where oil shale and the chemical industry are located) has been subjected to pollution with acidic compounds. In 1981–1988 the yearly mean nitrogen (N) deposition load was up to 11.1 kg ha. This N pollution level combined with the deposition of sulphur (S) could have seriously endangered the environment, but the simultaneous emission of strongly alkaline fly ash restrained acidification processes. After 1989–1991 the situation changed, and in 1994–1996 the N deposition load in northeastern Estonia remained within the range of 2.6 to 6.6 kg ha year and that of S within 2 to 50 kg ha year. Because the fly ash deposition is permanently decreasing, more sensitive lichens and mosses can be subjected to critical N+S loads in the future. The proportion of oil shale industry in total emission of NO_x_ in Estonia from stationary sources equals approximately 65 to 75%. During 1996–2000 the yearly mean concentration of NO_2_ in the air of towns increased from 9 to 12 to 16 to 29 μg m. The emission of N compounds was mainly caused by N oxides in flue gases from power plants, as well as ammonia and carbamide discharges from chemical plants. In 1988–1990 the estimated yearly total emission of NO_x_ (as NO_2_ equivalent) was about 18 to 18.6 thousand t and in 1994–2000, 9.9 to 11.8 thousand t.

## INTRODUCTION

The oil shale–based power industry has a strong impact on the ecosystems of the northeastern part of Estonia. The atmospheric influx of sulphur dioxide ($SO_2$), nitrogen oxides ($NO_x$), and alkaline fly ash as a result of combustion of oil shale in the power plants (PPs) is one of the main factors causing pollution in residential and rural areas. Construction of PPs operating on oil shale began in 1949 with the Kohtla-Järve PP and was finished in 1969 with the Estonian PP. The years 1975–1991, when annually 26 to 31 million t of oil shale were used, was the period of maximum emissions from PPs. Since 1998 the annual consumption of oil shale has been about 9 to 12 million t, which has considerably reduced pollutant emission[1].

The pollution load in northeastern Estonia results from simultaneous presence of acidic and alkaline components in the overground air layer. Alkaline fly ash has strongly prevented acidification processes in northeastern Estonia because increased basic cation deposition buffers fully acidic deposition and sheer alkalisation. The increase in atmospheric input of Ca-rich fly ash (simultaneously with $NO_x$ and $SO_2$) and alkaline precipitations in 1960–1990 caused important changes in environmental conditions and plant cover in bogs. For example, the *Sphagnum* cover in the Kurtna Landscape Reserve (see Fig. 1) is degraded[2]. In the period of 1984–1988 the pH of precipitations in some regions, regardless of high $SO_2$ and $NO_2$ concentrations, was as follows: 7.5 to 9.2 in the radius of 1.5 to 30 km from Estonian PP; 7.7 to 8.3 in the radius of 1 to 15 km from Kohtla-Järve PP, and 5.3 to 7.5 in the southern part of the study area (coast of Lake Peipsi)[3,4,5]. Later, in the years 1990–1998, the average pH of rainwater ranged between 6.4 and 7 in the Kohtla-Järve region[6] and between 6.3 and 6.9 in Jõhvi and the Kurtna Landscape Reserve[7,8]. It should be mentioned that in some other regions of Estonia the values of pH also showed a small decreasing trend during the last years[8]. In areas that are affected by high fly ash dust deposition during many years, remarkable signs of *Sphagnum* occurrence are noted after downfall of emissions. The effects of marked reduction (25 to 85%) of acidic and alkaline pollutant emissions on atmospheric chemistry and forest ecosystems have been studied also in some areas of East Germany[9].

Since 1960 the amounts and intensities of emissions as well as temporal changes and dynamics of oil shale fly ash and $SO_2$ in atmospheric air have been discussed in numerous papers[1,6,10,11,12]. Clearly systematised materials about $NO_x$ in the atmosphere and the deposition of N on the ground surface in northeastern Estonia have not yet been published. In addition to PPs, important sources of N emissions are the chemical plants (production of ammonia and carbamide) located in the town of Kohtla-Järve, as well as local lower-capacity, gas-fired boiler houses. The present paper deals with the specifics and temporal changes in air pollution with $NO_2$ and $SO_2$, as well as with the intensity of N and S deposition on the ground surface in the oil shale industry region in northeastern Estonia during the last 12 to 15 years.

## STUDY AREA AND METHODS

The study area (about 3360 km$^2$) is located in the northeastern part of Estonia (Ida-Viru County) between the Gulf of Finland and Lake Peipsi, bordering Russia in the east. The major pollution sources in this region are four PPs operating on oil shale: the Baltic PP and Estonian PP near the town of Narva, and the Kohtla-Järve PP and Ahtme PP (see Fig. 1). In addition to PPs, northeastern Estonia is also affected by emissions from the oil shale processing plant and the mineral fertiliser plant situated in the town of Kohtla-Järve.

The $SO_2$ and $NO_2$ concentrations in the atmospheric air were estimated using the numerical semiempirical atmospheric dispersion modelling method OND-86[13] based on Gaussian theory, as well as the modified variant of this method for the calculation of long-term mean concentrations[14]. The data on the air pollution level obtained by the automatic monitoring station "Opsis" in Kohtla-Järve for the years 1991–1996 were used together with the results of air sample analyses performed using standard methods[15] by the Virumaa Environmental Research Laboratory Ltd. at the stationary air sampling stations (in Kohtla-Järve, Jõhvi, and Narva). Temporal changes in deposition loads of N and S were analysed based on the results of measuring the $SO_4$-S and ($NO_3$+$NH_4$)-N loads in permanent snow cover in the winter period. The results of investigations and mapping the critical loads of N and S for terrestrial ecosystems in Estonia[16,17,18] and different environmental monitoring data[1,3,7,19] were also used. Yearly values of pollutant emissions were obtained from statistical data of enterprises.

## RESULTS AND DISCUSSION

### Emission Sources and Emissions

N air pollution in northeastern Estonia comes from two main sources: combustion of fuels (about 70%) and traffic (30%)[3,20]. In the last years the role of traffic has probably increased, but concrete factual data are not yet available. Emission of $NO_x$ from stationary industrial sources in northeastern Estonia is mainly from PPs operating on the local fossil fuel, oil shale. It constitutes about 90 to 95% of total $NO_x$ emission from industrial pollution sources in northeastern Estonia and approximately 65 to 75% of total emission of $NO_x$ from stationary sources in Estonia. In 1994 about 65% of total emission of $NO_2$ (42.5 thousand t) in Estonia were discharged by motor transport.

As the content of N in oil shale is relatively small (about 0.1%) and the combustion temperature in boilers is relatively low, the amount of $NO_x$ formed in the PPs is insignificant. The concentration of $NO_x$ in flue gases does not exceed 0.2 to 0.25 g m$^{-3}$ (as $NO_2$ equivalent)[21]. The total emission of $NO_2$ was greatest during the period of 1989–1991, when combustion of oil shale

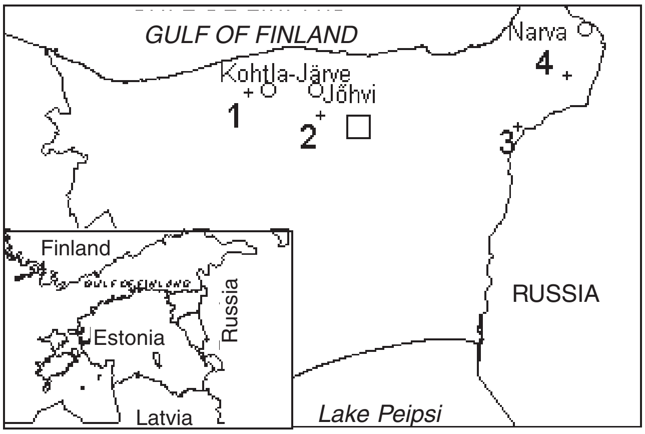

**FIGURE 1.** The study area: northeastern part of Estonia. The power plants (+): 1, Kohtla-Järve; 2, Ahtme; 3, Estonian; 4, Baltic. Box (¨ ) indicates Kurtna Landscape Reserve.

in PPs reached its peak with 24 to 27 million t/year, and the emission of $NO_2$ rose to the level of 14.5 to 16.5 thousand t (together with other sources totalling up to 18.6 thousand t). Since 1994, due to the reduction in the production of electrical energy, the yearly emission of $NO_2$ from PPs has stabilised on the level of 9.2 to 10.8 thousand t (Table 1). $NH_3$ and carbamide are emitted from the mineral fertiliser plant in Kohtla-Järve. In 1990–2000, $NH_3$ and carbamide discharge decreased essentially by about 5 to 6 times (Table 1).

Tables 1 and 2 show the changes in the emission of N compounds ($NO_x$, $NH_3$, carbamide), $SO_2$, and alkaline oil shale fly ash in northeastern Estonia during the last 12 years. The role of $NO_2$ and $SO_2$ in emissions rose from 1.1 in 1988 to 1.9 in 2000. In some regions, affected by local PPs, and oil shale processing and chemical plants of the town of Kohtla-Järve (Table 2), the ratio of emitted $NO_2$+$SO_2$ to alkaline dust increased markedly after 1988 due to installation of electric filters in the PPs, reaching 28 to 36 in 1996–2000 because of reduced capacity of PPs.

Until 1988 the fly ash emission in northeastern Estonia was high, which caused important changes in the chemical composition of bog water (the pH value rose from 2 to 3, to 5 to 6) and in plant cover, etc.[2], whereas no acidification took place. Since 1989–1991, the situation with atmospheric air pollution has changed: the proportion of acidic components ($SO_2$, $NO_2$) in emission compared to alkaline fly ash has increased considerably[10].

## Dynamics of Air Pollution

Investigations of the dynamics of air pollution with N compounds (see Table 3) carried out in 1991–2000 in the surroundings of local PPs and chemical plants in Kohtla-Järve showed that the maximum values of $NO_2$ short-term concentrations ($C_m$, measured during 30 min) were in the range of 79 to 779 µg $m^{-3}$, with maximum values of daily means ($C_d$) in the range of 26 to 130 µg $m^{-3}$ and a yearly average ($C_y$) of 9 to 29 µg $m^{-3}$.

The $NO_2$/NO ratio in the air of the towns of Narva and Kohtla-Järve (see Fig. 1) was approximately 1:(1-1.1), which means that the actual total concentration level of $NO_x$ is higher than measured by air monitoring for $NO_2$[1]. The increase in the $NO_2$ air pollution level (see Table 3, annual mean) in towns was probably caused by heavy traffic in the last years. Comparatively high concentrations ($C_m$ up to 633 µg $m^{-3}$ and $C_y$ 26 to 32.5 µg $m^{-3}$ in 1999–2000) of $NO_2$ were fixed in Narva, where traffic emissions are higher compared to other towns in northeastern Estonia.

With respect to forest trees, the following critical loads were recommended[22] for the ambient air:

**TABLE 1**
**Emission of N Compounds into the Atmosphere from Industrial Stationary Sources in Northeastern Estonia from 1988 to 2000 (thousand t)[1]**

| Pollutants | 1988 | 1990 | 1992 | 1994 | 1996 | 1998 | 2000 |
|---|---|---|---|---|---|---|---|
| $NO_x$ (as $NO_2$) | 18.6 | 18.1 | 13.9 | 10.6 | 11.8 | 10.2 | 9.92 |
| *– among this from PP* | *16.1* | *16.5* | *12.7* | *9.2* | *10.8* | *9.3* | *9.3* |
| $NH_3$ | – | 0.54 | 0.34 | 0.22 | 0.16 | 0.11 | 0.10 |
| Carbamide | – | 0.84 | 0.40 | 0.61 | 0.28 | 0.09 | 0.15 |
| *N compounds in total* | *18.6* | *19.5* | *14.6* | *11.4* | *12.2* | *10.4* | *10.2* |
| $SO_2$ | 200 | 218 | 150 | 114 | 96.6 | 84.4 | 85.1 |
| Fly ash (FA) | 195 | 182 | 133 | 87.1 | 74.4 | 70.0 | 49.7 |
| *Ratio of ($NO_2$+$SO_2$)/FA* | *1.1* | *1.3* | *1.2* | *1.4* | *1.5* | *1.4* | *1.9* |

**TABLE 2**
**Emission of Acidic Compounds and Alkaline Fly Ash in the Region of Kohtla-Järve from 1988 to 2000 (thousand t)[1,10]**

| Pollutants | 1988 | 1990 | 1992 | 1994 | 1996 | 1998 | 2000 |
|---|---|---|---|---|---|---|---|
| $NO_x$(as $NO_2$) | 1.12 | 0.82 | 0.70 | 0.59 | 0.37 | 0.49 | 0.50 |
| $NH_3$ | – | 0.37 | 0.28 | 0.14 | 0.07 | 0.06 | 0.04 |
| $SO_2$ | 12.9 | 11.2 | 7.9 | 7.9 | 9.1 | 7.0 | 4.3 |
| $H_2S$ | – | 0.019 | 0.016 | 0.011 | 0.012 | 0.008 | 0.009 |
| Fly ash (FA) | 36.2 | 1.45 | 1.4 | 0.60 | 0.28 | 0.21 | 0.17 |
| *Ratio of ($NO_2$+$SO_2$)/FA* | *0.39* | *8.3* | *6.1* | *14.2* | *33.6* | *35.6* | *28.0* |

**TABLE 3**
**Concentrations of Acidic Pollutants in the Town of Kohtla-Järve by Air Monitoring in 1991 to 2000 (C, μg m$^{-3}$)[1]**

| Pollutant | C | 1991 | 1992 | 1993 | 1994 | 1995 | 1996 | 1997 | 1998 | 1999 | 2000 |
|---|---|---|---|---|---|---|---|---|---|---|---|
| $NO_2$ | $C_m$ | 118 | 85 | 84 | 87 | 79 | 139 | 285 | 243 | 779 | 377 |
| | *$C_d$ | 42 | 26 | 35 | 29 | 31 | 70 | 71 | 63 | 130 | 66 |
| | $C_y$ | 9.5 | 9.0 | 10 | 11 | 12 | 16 | 21 | 19 | 29 | 27 |
| $NH_3$ | $C_m$ | – | – | 170 | 380 | 204 | 185 | 85 | 475 | 190 | 186 |
| | *$C_d$ | – | – | – | – | 26.5 | 17 | – | – | – | 102 |
| | $C_y$ | – | – | 14 | 20 | 11 | 8.1 | 11 | 8.3 | 18 | 27 |
| $SO_2$ | $C_m$ | 442 | 342 | 348 | 270 | 443 | 394 | 315 | 530 | 256 | 179 |
| | *$C_d$ | 108 | 106 | 113 | 73 | 116 | 81 | 117 | 315 | 100 | 74 |
| | $C_y$ | 19 | 16 | 17 | 12 | 12 | 17 | 23 | 19 | 23 | 21 |

* Maximum values of daily means.

- For $NO_x$ (as $NO_2$ equivalent), 30 μg m$^{-3}$ as annual mean and 95 μg m$^{-3}$ as 4-h mean; it is assumed that $SO_2$ load is close to its critical level.
- For $SO_2$, 20 μg m$^{-3}$ as annual mean or half-year mean (October–March) for winter conditions. In addition, 10 μg m$^{-3}$ as annual mean is recommended to protect sensitive lichen species.
- For ammonia[22], an annual mean of 8, a monthly mean of 23, a daily mean of 270, and a 1-h maximum of 3300 μg m$^{-3}$.

Table 3 shows that during 1995–2000 the annual mean concentration of $SO_2$ in the air increased slightly in the region of Kohtla-Järve and remained within the range of 17 to 23 μg m$^{-3}$, which is close to the critical level for forest trees and lichen. At the same time, the annual mean concentration of $NO_2$ increased from 9 to 12 μg m$^{-3}$ in 1991–1995 to 21 to 29 μg m$^{-3}$ in 1997–2000. In 1999–2000 the annual average concentration of ammonia jumped to 18 to 27 μg m$^{-3}$, which is more than recommended for vegetation. This tendency shows that a continuing reduction in emissions from PPs of the alkaline component (fly ash) and increase in the role of $NO_2$ and $SO_2$ in emissions (Tables 1 and 2) may in the future cause negative effects on growth parameters of trees and acidification processes in the environment. This may be one reason why the condition of the conifers (pine, spruce) in the surroundings of Kohtla-Järve[6] has not yet improved, regardless of the decrease in the amount of pollutants, including organic compounds, emitted annually in 1990–1994 (see Table 2).

## Deposition Loads of N and S on Ground Surface

Deposition loads of N ($C_N$) and S ($C_S$) on ground surface and their acidifying impact have changed temporally in accordance with decreasing emissions of $SO_2$ and $NO_x$ (Tables 1 and 2).

By mapping the ecosystems in northeastern Estonia, the following critical loads for N and S (kg ha$^{-1}$ year$^{-1}$) have been established[16,17,18] to prevent acidification in this region (the ranges of variation for the entire Estonian territory are shown in brackets):

- Maximum load for sulphur $CL_{max}(S)$ = 33 (25–35);
- Minimum load for nitrogen $CL_{min}(N)$ = 4.9 (3.6–4.9), whereas $C_S = CL_{max}(S)$;
- Maximum load for nitrogen $CL_{max}(N)$ = 67 (57–67), whereas $C_S = 0$;
- Critical load for eutrophication (nutrient nitrogen) $CL_{nut}(N)$ = 4.9 (4.9–6.4).

The estimated critical load of N for ombrotrophic bogs is 5 to 10 kg ha$^{-1}$ year$^{-1}$; a higher deposition load would cause the decrease of peat mosses (*Sphagnum*)[23]. The following values are critical: for calcareous species-rich grasslands, 14 to 25 kg N ha$^{-1}$ year$^{-1}$; for coniferous trees (nutrient imbalance), 10 to 15 kg N ha$^{-1}$ year$^{-1}$[16].

Table 4 displays the pollution loads of N and S in northeastern Estonia for the period of 1981–1996 determined by monitoring (snow sampling) and computed data. Comparison of actual deposition loads ($C_N$ and $C_S$) with the above critical loads shows that in the years 1985–1990 the deposition load of S ($C_S$ = 33.7 to 57 kg ha$^{-1}$ year$^{-1}$) noticeably exceeded the corresponding critical level $CL_{max}(S)$ for the northeastern Estonian area, and the deposition load of N in 1981–1989 exceeded the critical value of acidic N $CL_{min}(N)$ ($C_N > CL_{min}(N)$ contemporary with $C_S > CL_{max}(S)$ in the Jõhvi–Kurtna Landscape Reserve. The critical load for eutrophication (nutrient N), $CL_{nut}(N)$, was exceeded ($C_N$ = 6.4 to 11.1 kg ha$^{-1}$ year$^{-1}$) by up to 2.2 times in the years 1981–1994, which is also mentioned in the calculation of critical loads[16,17].

Tables 4 and 5 show that during the period of 1981–1994 the reduction of N and S deposition was necessary, but the simultaneous presence of strongly alkaline oil shale fly ash in emis-

**TABLE 4**
**Yearly Mean Deposition Loads[1,3,7,19] of N ($C_N$) and S ($C_S$) in Northeastern Estonia in 1981–1996 (kg ha$^{-1}$ year$^{-1}$)**

| Location (see Fig. 1) | C | 1981 | 1985 | 1988 | 1989 | 1990 | 1994 | 1995 | 1996 |
|---|---|---|---|---|---|---|---|---|---|
| Jõhvi–Kurtna | $C_N$ | 6.4 | 8.6 | 11.1 | 7.2 | 2.5 | 6.6 | 3.9 | 2.6 |
| Landscape Reserve | $C_S$ | 31.4 | 49.4 | 57.0 | 33.7 | 44.6 | 27.5 | 22 | 19 |
| Kohtla-Järve | $C_N$ | – | – | 1.5 | – | – | – | – | 3.8 |
| | $C_S$ | | 45 – 200 | | – | 7.3[2] | | 9.7 – 50 | 9.7 |
| | | | | | | | | | 2 – 7[2] |
| Southern part | $C_N$ | – | – | 3.2[1] | – | – | | – | – |
| (Lake Peipsi) | $C_S$ | | 3 – 41[2] | | – | – | | 2 – 10 | 1.8 – 4[2] |

[1] In winter 1986/1987.
[2] Computed data for 1996–1998.

**TABLE 5**
**LRequirements for the Reduction of N ($C_N$) and S ($C_S$) Deposition Depending on Their Actual Deposition Loads[23]**

| $C_N$ | $C_S$ | Reduction Requirements |
|---|---|---|
| $=CL_{nut}$ (N); $=CL_{max}$(N) | $=CL_{max}$(S) | N and S deposition reductions are interchangeable |
| $<CL_{nut}$(N); $<CL_{max}$(N) | $>CL_{max}$(S) | S deposition reduction is mandatory |
| $>CL_{nut}$(N); $>CL_{max}$(N) | $<CL_{max}$(S) | N deposition reduction is mandatory |
| $>CL_{nut}$(N); $>CL_{max}$(N) | $>CL_{max}$(S) | N and S depositions reduction are mandatory |
| $<CL_{nut}$(N); $<CL_{max}$(N) | $<CL_{max}$(S) | No deposition reduction is required |

sions and atmospheric air, causing the high base cation ($Ca^{2+}$) deposition of 20 to 750 kg ha$^{-1}$ year$^{-1}$ in the Jõhvi, Kohtla-Järve, and Narva areas (see Fig. 1), prevented acidification processes and sheer alkalisation in northeastern Estonia[1,11]. By 1998, deposition of $Ca^{2+}$ decreased in most of northeastern Estonia to the level of 5 to 30 kg ha$^{-1}$ year$^{-1}$.

Also, Table 4 shows that the deposition loads of N and S decreased noticeably after a considerable decrease in emissions of $NO_2$ and $SO_2$ in 1989–1991 (see Table 1), and after 1995 they did not exceed their critical values for northeastern Estonia. A comparison of depositional data in Table 4 with the reduction requirements given in Table 5 indicates that no deposition reduction is required at the present time, but if N deposition increases further, its load will exceed CL values and the extra N might start to influence the plant communities in bogs[17].

## CONCLUSIONS

The comparatively high deposition loads of N and S in northeastern Estonia before 1994 have not caused the acidification of ecosystems because of the simultaneous presence of the alkaline pollution component (Ca-rich oil shale fly ash) in the environment. After 1995 the deposition loads of N and S on the ground have not clearly exceeded the critical levels for acidification and their reduction is not required. Yearly mean concentrations of $NO_2$, $NH_3$, and $SO_2$ in the atmospheric air of towns and their surroundings are at the limit level or exceed critical levels for trees (conifers, pine) in some regions of northeastern Estonia. These concentrations need special attention because if the decrease in the emission of fly ash continues, the sensitive lichens and mosses, as well as conifers in some more polluted areas, may be affected by $NO_2$ and $SO_2$ in the future.

## ACKNOWLEDGEMENTS

This research was supported by grant No. 3776 of the Estonian Science Foundation.

| | | |
|---|---|---|
| Received: | July | 27, 2001 |
| Revised: | October | 8, 2001 |
| Accepted: | October | 15, 2001 |
| Published: | November | 21, 2001 |

## BIOSKETCH

**Valdo Liblik, Ph.D.,** is a senior scientist and head of the North-East Estonian Department of the Institute of Ecology at the Tallinn University of Educational Sciences (Estonia). His research interests include pollutants' transmission processes in the atmosphere, impact of different environmental factors on ecosystems and landscapes, and environmental protection. He has conducted research in north-eastern Estonia, in the area of oil shale mining and processing. He is laureate of the Science Award of the Republic of Estonia in the fields of bio-, geo-, and agricultural sciences.
